# Prenatal exposure to testosterone (2D:4D) and social hierarchy together predict voice behavior in bankers

**DOI:** 10.1371/journal.pone.0180008

**Published:** 2017-06-28

**Authors:** Erik Bijleveld, Joost Baalbergen

**Affiliations:** 1 Behavioural Science Institute, Radboud University, Nijmegen, The Netherlands; 2 Department of Psychology, Utrecht University, Utrecht, The Netherlands; University of Vienna, AUSTRIA

## Abstract

*Prohibitive voice behaviors* are employees’ expressions of concern about practices, incidents, or behaviors that may potentially harm the organization. In this study, we examined a potential biological correlate of prohibitive voice: prenatal exposure to testosterone. In a sample of bankers, we used 2D:4D (i.e., the ratio of the length of the index finger to the length of the ring finger) as a marker for prenatal exposure to testosterone (lower 2D:4D suggests higher prenatal exposure to testosterone). We used a self-report scale to measure prohibitive voice. For low-ranked employees, lower 2D:4D was related to using less voice. No such relation was found for high-ranked employees. Conclusions should be drawn with caution, because the findings only applied to voice regarding the organization as a whole (and not to voice regarding the own team), and because of methodological limitations. However, the findings are consistent with the ideas that (a) people low in 2D:4D tend to strive to attain and maintain social status and that (b) remaining silent about perceived problems in the organization is—at least for low-ranked employees—a means to achieve this goal.

## Introduction

In the past years, the financial sector has been plagued by financial scandals, often originating in misbehaviors of some individual employees. To improve their procedures, financial institutions currently try their best to encourage employees to speak out when they perceive practices, incidents, or behaviors that may potentially harm the organization—i.e., they encourage employees to use *prohibitive voice* [1]. In the present study, we will examine prohibitive voice in a natural setting. Specifically, in a sample of bankers, we will explore a potential biological correlate of prohibitive voice: prenatal exposure to testosterone. By taking an interdisciplinary approach to voice, we provide a demonstration of how neurobiological insights can inspire new perspectives on organizational behavior. In the long run, this research may help us to better understand why some employees speak up, while others do not.

### Prohibitive voice

In management science, *voice* is broadly defined as “discretionary communication of ideas, suggestions, concerns, or opinions about work-related issues with the intent to improve organizational or unit functioning” (p. 375 of [2]). *Prohibitive voice* is a subtype of voice, involving only expressions of concern about potential harm to the organization [1]. Although constructive in its intent, prohibitive voice is always a challenge to the status quo: employees who use prohibitive voice convey that they feel that the organization needs to change [1–4].

An important goal for previous research has been to understand how employees decide whether to use voice. To address this issue, the *expectancy model of voice* [2] proposes that voice results from an a priori, mental weighting of costs and benefits of potential outcomes. In essence, this model suggests that the strength of employees’ *motive* to improve the organization is the driving force behind voice. Importantly, though, the expectancy model also suggests that strong motives to improve the organization cause voice only in some conditions. Specifically, employees’ cost–benefit weightings take into account assessments of *efficacy* (employees use voice more if they expect this will actually have an effect) and *safety* (people use voice more if they expect they will not incur personal harm).

The expectancy model of voice can explain a large amount of data. For example, conscientious employees [5] and committed employees [6] (strong motive to improve the organization), use more voice. Employees who expect their manager to take their concerns seriously [3] (high efficacy), use more voice. In organizations in which employees feel supported [7] (high safety), employees use more voice. Employees higher in the hierarchy [8–10] and employees who have good alternative job opportunities [11] (high safety), use more voice.

To connect the literatures on voice and testosterone (see next section), we highlight two observations here. First, it is reasonable to conceptualize the decision to use voice as a decision under risk [12]. That is, when an employee chooses not to use voice, the status quo is not challenged and the predictability of the future does not change. However, when an employee chooses to use voice, this creates new, possible, personal outcomes that may either be positive (e.g., getting recognition for helping the organization [2]) or negative (e.g., hurting one’s reputation, getting fired [13]). Regardless, due to the creation of more possible outcomes, the predictability of the future decreases. Arguably, predictability of future outcomes will especially decrease (after using voice) for employees who are lower in rank. After all, almost by definition, lower-ranked employees have less control over decision-making processes in their organization [14]. Thus, their future outcomes depend more strongly on others, rather than on themselves, causing their outcomes to become less predictable to them. So, especially for low-rank employees, to use voice is like choosing the risky option in a gamble.

Second, it is possible to think of voice as a potential threat to one’s place in the social hierarchy. As reflected in the expectancy model [2], a key concern that employees have is that using voice may lead to severe, negative, personal consequences. Systematic interviews with employees [13,15], for example, suggest that voice is associated with fears of getting fired, of falling out of favor, of being labelled a complainer, of being ostracized, of being accused of being a snitch, and so forth. Relevant for the present purposes, these potential consequences all involve a loss of social standing. Also here, due to their lesser control over decision-making processes in their organization, negative consequences are looming especially for employees who depend strongly on decisions by their superiors [13,15]. So, especially for low-rank employees, the decision to use voice involve threats to their place in the social hierarchy.

### Organizing effects of testosterone

Testosterone is a gonadal hormone that contributes to a wide range of behaviors, such as behaviors related to aggression [16], caregiving [17], competition [18], sexuality [19], and trust [20] (but see [21]). Related to the previous section, we suggest that two lines of research related to testosterone are potentially relevant for understanding the biological processes that underlie decisions to exhibit voice. These lines of research pertain to risk taking and social status, respectively. We will discuss these lines of research in turn. In this introduction, we focus mainly on research on the organizing effects of prenatal exposure to testosterone (and less so on the effects of circulating testosterone).

Research on the organizing effects of testosterone has mainly relied on measurements of the ratio of the second and the fourth digit (or 2D:4D). Indeed, research suggests that 2D:4D is correlated with exposure to testosterone in utero. For example, newborns [22] and children [23] who had been exposed to more testosterone before birth (e.g., in the amniotic fluid), had smaller 2D:4D. Also, people with Congenital Adrenal Hyperplasia (who had been exposed to supra-normal levels of testosterone in utero) had smaller 2D:4D than controls [24] (but see [25]), while people with Klinefelter’s syndrome (who had been exposed to sub-normal levels of testosterone in utero) had larger 2D:4D [26]. Finally, mice whose mothers received testosterone during pregnancy, were born with smaller 2D:4D [27]. These findings suggest that 2D:4D can be used as a marker variable: smaller 2D:4D suggests greater prenatal exposure to testosterone (but see Discussion).

#### 2D:4D and risk taking

Several studies have examined the relationship between 2D:4D and economic risk taking. Typically, in these studies, participants repeatedly choose between gambles that involve various levels of risk [28]. Some of these studies show that people low in 2D:4D have a greater preference for risky options [28,29]. At the same time, several studies with similar designs show null effects [28,30]. So, there is currently no straightforward evidence that 2D:4D relates to economic risk taking [28].

Still, for two reasons, it is too soon to discard this hypothesis altogether. First, while the evidence from experimental gambles is weak, 2D:4D does correlate with various real-life behaviors that can be interpreted to be risky [12,31]. For example, a survey study showed that, at least in males, 2D:4D is related to behaviors such as “engaging in dangerous sports (e.g. mountain climbing or sky diving)” and “shoplifting a small item (e.g. a lipstick or pen)” [31]. Similarly, people with low 2D:4D are more likely to prefer risky career paths [32] and financial traders low in 2D:4D choose more risky options on the trading floor [33]. So, people low in 2D:4D seem more likely to select real-life actions that involve risk. Second, as associations between 2D:4D and behavior are context-dependent [34–36], it seems over-ambitious to expect a general association between 2D:4D and risk-taking behavior [37]. One study, for example, showed that 2D:4D was related to risk-taking (measured with a gambling task), but only among participants who had just recalled an incident in which they were low in power [36]. So, taken together, it may be true that prenatal exposure to testosterone prepares people to prefer risky options later in life, at least in some contexts [37].

Considering these prior findings, and considering that voice can be seen as a risk-taking behavior especially for low-ranking employees, we hypothesize that:

*Hypothesis 1: For low-rank (vs. high-rank) employees, lower 2D:4D should relate to a greater tendency to use voice*.

#### 2D:4D and social status

Another line of research suggests that people low (vs. high) in 2D:4D are more inclined to try to attain and maintain high social status. This idea is supported by two classes of findings. First, low 2D:4D is associated with the attainment of high social status, both in humans [38–40] and in other animals [41]. In humans, for example, low 2D:4D is associated with success in various types of sports [38], education [39], and careers [40]. So, speculatively, prenatal testosterone prepares people to use behavioral strategies (e.g., making certain career choices) that allows them to outperform their peers [42]. Second, in behavioral experiments, people low in 2D:4D are often found to respond strongly to cues that are related to social status. For example, they were less willing to accept unfair offers from others [43], they responded with more aggression after watching an aggressive video [34], and they allocated less resources to other people who had a dominant-looking face [35]. These cues have in common that they signal status-related threats; people low in 2D:4D thus seem extra sensitive to these.

Together, this research implies that people low in 2D:4D care about attaining and maintaining social status, and that they are typically successful in this endeavor. Considering this implication, and considering that low-ranking employees can protect their social status by *not* using voice, we hypothesize that:

*Hypothesis 2: For low-rank (vs. high-rank) employees, lower 2D:4D should relate to a lesser tendency to use voice*.

In the present study, we will test our two competing hypotheses in a sample of employees of a large financial institution. We will measure 2D:4D and self-reported prohibitive voice [1]. We will examine two types of prohibitive voice behaviors: voice about problems seen in the own team and voice about problems seen elsewhere in the organization. We further administered two questionnaires that were unrelated to the present hypotheses [44,45]. Data from these questionnaires are included in the supplementary information (S1 and S2 Files), but we will not present analyses in this paper. Besides testing our hypotheses, we will present analyses of sex differences, as prior work revealed sex differences in both 2D:4D [46] and voice [3].

## Methods

### Ethics statement

The study was conducted in compliance with Dutch law. Specifically, as our study did not use invasive techniques (e.g., drug administration, blood sampling) and as it was conducted among healthy and consenting adults, the Medical Research (Human Subjects) Act did not apply. Accordingly, our study was not eligible for evaluation by a registered Medical Ethics Committee. Instead, as is the standard procedure for self-report and behavioral studies conducted in the Netherlands, we followed the Code of Ethics from the Dutch Association of Psychologists (NIP). The study was approved by the local faculty board (Utrecht University, Social Sciences) and by the 'ethics team' of the organization where the study was conducted. The latter committee also approved the digital consent procedure, in which participants learned that their responses would be processed anonymously, and that they were free to quit without giving a reason if they did not want to participate anymore. All participants indicated their understanding of this information and gave consent by clicking a button in the digital survey environment. Both hypotheses were conceived before data collection as reported in this paper. We report all exclusions and all measures.

### Participants and procedure

This research was conducted among 100 employees of a major financial institution in the Netherlands. Employees worked in various departments within the institution (e.g., they were involved in risk grading, private banking, public affairs, and economic research). After a brief explanation of the purpose of the research, participants learned that the research consisted of two parts. First, participants would receive a link to an online self-report survey via e-mail. Second, an appointment was made with participants to make a scan of the right hand of the participant, enabling us to measure 2D:4D.

Twenty participants were excluded from analysis because we were unable to acquire a scan of their hand (most often, due to their busy schedules). Nine further participants were excluded because they did not (fully) complete the questionnaire. This resulted in a final sample of 71 participants (38 men, 33 women; M_age_ = 46, SD_age_ = 9.7).

### Measures

Unless otherwise noted, participants responded to all survey items on seven-point Likert-type scales ranging from 1 (fully disagree) to 7 (fully agree).

#### Prohibitive voice

Prohibitive voice behaviors within the own team were measured using the prohibitive voice self-report scale by Liang et al. [1]. This scale consists of five items. Example items are “I dare to point out problems when they appear in the work unit, even if that would hamper relationships with other colleagues”. Prohibitive voice behavior concerning problems noticed elsewhere in the organization were measured using the same items, with the words “work unit” replaced by “the organization” or “in another organizational unit” (e.g. “I speak up honestly with problems in another organizational unit that might cause serious loss to the organization, even when dissenting opinions exist”). Both the voice within team scale (α = .87) and the voice within organization scale (α = .89) had good reliability.

#### Hierarchy

Participants self-reported their place in the organizational hierarchy on a ten-point scale ranging from 1 (Bottom) to 10 (Top). Literally, the question we asked was as follows: “[the organization] has a hierarchical structure. Please indicate your place in this hierarchy.”

#### 2D:4D ratio

2D:4D was determined from a scan from the inside of the right hand, because right-hand digit ratios may be more sensitive to prenatal androgens [46]. The length of both the index finger (2D) and ring finger (4D) were defined as the distance between the middle of the tip of the finger and the middle of the crease most proximal to the palm. Handprints were measured by the second author and an independent rater, using SmallMeasure software. Measurements displayed high inter-rater correlation, *r* = .89, p < .001. The average of both raters’ measurements was used in all analyses.

## Results

### Descriptive statistics

On average, participants rated their place in the organization’s hierarchy at about the midpoint of the scale (*M* = 5.2, *SD* = 2.1). People rated their tendency to use voice, on average, on the higher half of the scale (voice within team, *M* = 5.3, *SD* = 1.1; voice within organization, *M* = 4.7, *SD* = 1.3). Average 2D:4D was similar to other samples of healthy adults (*M* = 0.96, *SD* = .03), and did not differ much between men (M = 0.95) and women (M = 0.96). We report correlations between these variables in Table 1.

**Table 1:** Correlations between the main variables of interest.

	1	2	3	4
1. Place in the hierarchy	1			
2. 2D:4D	-.10	1		
3. Voice (team)	.02	.01	1	
4. Voice (organization	.28*	.00	.42**	1

*p < .05;

**p < .01.

### 2D:4D and voice

To test our hypotheses, we performed a general linear model analysis with voice within team and voice within organization as dependent variables, and 2D:4D and place in the hierarchy (henceforth: hierarchy) as independent variables.

**Fig 1:**
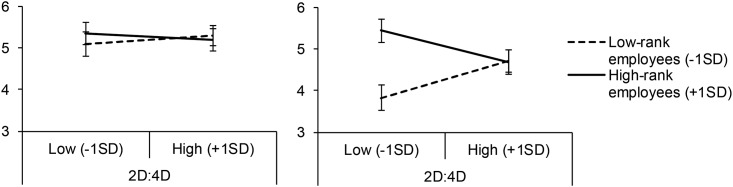
Estimated means of people’s tendency to use voice, as a function of 2D:4D and rank within the organizational hierarchy. Please note that these estimates were computed based on the general linear model described in the main text (so, not by splitting the dataset into two groups). Left panel: voice within team. Right panel: voice within organization. Error bars represent standard errors around the estimate.

For voice within team, no significant main effect of 2D:4D was found, *F*(1, 67) < .1, *p* = .915, β = .01, η^2^_p_ < .01. Neither, there was a significant main effect of hierarchy, *F*(1, 67) < .01, *p* = .811, β = .03, η^2^_p_ < .01. The interaction between 2D:4D and hierarchy was not significant either, *F*(1, 67) = 0.4, *p* = .519, β = -.09, η^2^_p_ < .01. So, for voice within own team, neither hypothesis was supported by the data. Estimates are presented in Fig 1, left panel.

For voice within organization, there was no significant main effect of 2D:4D, *F*(1, 67) < .01, *p* = .813, β = .03, η^2^_p_ < .01. However, there was a main effect of hierarchy, *F*(1, 67) = 7.9, *p* = .006, β = .32, η^2^_p_ = .11. This effect indicated that higher-status employees were more inclined to use voice behaviors. Importantly, as predicted by both hypotheses, there was a significant interaction between 2D:4D and hierarchy, *F*(1, 67) = 7.2, *p* = .009, β = -.32, η^2^_p_ = .10. To directly test our hypotheses, we estimated the effect of 2D:4D separately for people low (–1SD) vs. high (+1SD) in the hierarchy (Fig 1, right panel, see [47]). Simple slope analyses indicated that, for low-rank employees, 2D:4D was positively related to voice, β = .35, *t*(67) = 2.1, *p* = .037. In line with Hypothesis 2, but not Hypothesis 1, this finding suggests that low-rank employees with lower 2D:4D (indicating high prenatal exposure to testosterone) are inclined to use less voice. For high-rank employees, 2D:4D was negatively and not significantly related to voice, β = -.30, *t*(67) = 1.7, *p* = .078.

For the sake of completeness, again using simple slopes analysis, we also estimated the effect of hierarchy separately for participants with a low (–1SD) vs. high (+1SD) 2D:4D. For low-2D:4D participants, place in the hierarchy strongly predicted voice, β = .64, *t*(67) = 3.7, *p* < .001. For high-2D:4D participants, hierarchy did not predict voice, β = .00, *t*(67) = .0, *p* = .981.

To examine whether the interaction between 2D:4D and hierarchy still surfaced after controlling for potentially confounding variables, we added sex, age, tenure (i.e., number of years at the company), and education level (i.e., one dummy variable indicating undergraduate degrees, one dummy variable indicating graduate degrees) to the model as control variables. We should note that three participants did not report the number of years they worked at the company and that one participant did not report their age. As a result, this analysis was performed with N = 67. No significant effects were found for age, tenure, and education level, *F*’s(1, 58) ≤ 2.4, *p*’s ≥ .134, β’s ≥ -.40, β’s ≤ .12, η^2^_p_ ≤ .04. The main effect of sex was significant, *F*(1, 58) = 8.9, *p* = .004, β = -.71, η^2^_p_ = .13, with men self-reporting more voice than women [3]. Like before, no significant main effect for 2D:4D was found, *F*(1, 58) = .2, *p* = .680, β = .05, η^2^_p_ < .01. However, the main effect for hierarchy was significant, *F*(1, 58) = 4.4, *p* = .040, β = .25, η^2^_p_ = .07). In further support of Hypothesis 2, the interaction between 2D:4D and hierarchy was still significant after controlling for the potential confounders, *F*(1, 58) = 5.1, *p* = .027, β = -.26, η^2^_p_ = .08.

### Sex differences

We further explored the effects of sex. We conducted a general linear model analysis, with voice in the organization as a dependent variable, and hierarchy and 2D:4D as independent variables. To examine how sex interacts with 2D:4D and hierarchy, we added sex as an independent variable. Like before, a significant main effect of sex was found [3], with men speaking up more than women, *F*(1, 64) = 6.6, *p* = .013, β = -.58, η^2^_p_ = .09. The interaction between 2D:4D and hierarchy was again significant, *F*(1, 64) = 5.9, *p* = .018, β = -.28, η^2^_p_ = .09. However, sex did not significantly interact with 2D:4D, *F*(1, 64) < .1, *p* = .853, β = .04, η^2^_p_ < .01, nor with hierarchy, *F*(1, 64) = .8, *p* = .383, β = .20, η^2^_p_ = .01. When we added the three-way interaction of sex, 2D:4D, and hierarchy to the model, this interaction was not significant, *F*(1, 63) = .3, *p* = .621, β = -.13, η^2^_p_ < .01. These findings thus provide no evidence for any sex differences in how hierarchy and 2D:4D together predict voice.

## Discussion

In our study, we found that lower 2D:4D (suggesting higher prenatal exposure to testosterone) was associated with less self-reported voice, particularly among low-ranked employees. Thus, results are consistent with the ideas that (a) employees low in 2D:4D strive to attain and maintain social status [38–40] and that (b) *not* using voice is a way of achieving this goal [2,13,15], at least for low-ranked employees. In other words, speculatively, the results suggest that low-ranked employees low in 2D:4D are not willing to risk the potential adverse social outcomes of voice; thus, they are less likely to speak out when they see problems in their organization. This finding supports Hypothesis 2, but not Hypothesis 1.

Importantly, this effect was only found for prohibitive voice about problems seen within the organization as a whole, but not for voice about problems within the own team. Though post-hoc, an explanation for this null effect may lie in the fact that hierarchical status between team members is relatively small. So, in team contexts, exerting voice may involve less threats to one’s social status. It may therefore be the case that people may decide to use voice in most cases (see Fig 1, left panel), regardless of 2D:4D.

Combining ideas from neuroendocrinology and management science, the present research suggests that voice is underpinned by, or at least modulated by, the testosterone system. This suggestion opens up new ways of thinking about voice. More concretely, the expectancy model of voice [2] suggests that people make an a priori cost–benefit computation before they decide (not) to use voice. The present study suggests that these cost–benefit computations can be biased by testosterone. So, when trying to understand and predict who will use voice and who will not, the testosterone literature (and the 2D:4D literature in particular) may give valuable, new information. For example, very speculatively, it suggests that employees who usually perform well (associated with low 2D:4D [40]) and employees who usually act dominantly in confrontations (associated with low 2D:4D [34]) may counterintuitively *not* be the people who will speak out, especially when they are also low in rank. Of course, these proposed correlations may turn out to be weak or even nonexistent, but they provide an example of how research on testosterone can be used to further inform management science [33,40].

### Alternative interpretations

Our hypotheses were based on known associations between 2D:4D and two specific behavioral tendencies (i.e., to take risks, and to strive for social status). However, 2D:4D is associated with various other behavioral tendencies as well, some of which are potentially relevant to voice. Thus, alternative interpretations of our data are possible. Here we discuss two of these, drawing from prior findings.

First, people low in 2D:4D have been found to be more likely to reject unfair options in an ultimatum game (at least in neutral circumstances) [43]. That is, in this prior study [43], people low in 2D:4D rather forewent money, than to take deals that were unfair to them. This prior finding may be taken to suggest that people lower in 2D:4D are especially sensitive to unfairness. Thus, speculatively, people lower in 2D:4D may be particularly likely to fear unfair treatment from their organization, perhaps particularly when they are lower-ranked. For this reason, they may be more likely to decide to not use voice. This interpretation is related to our social status-related reasoning in the introduction (which led to hypothesis 2), but in addition, it suggests that people’s perceptions of (un)fairness may also contribute to their decisions to use voice.

Second, people low in 2D:4D have been found to act cooperatively, rather than egoistically or altruistically, in a public goods game [48]. That is, in this prior study [48], people low in 2D:4D tended to contribute exactly as much as was needed to serve the common cause, rather than more or less. This finding may be taken to suggest that people low in 2D:4D attain high social status by acting normatively (i.e., they cooperate, which helps preserve their status; but they do not do more than necessary, which helps prevent them from being exploited by others). In work settings, *not* using voice may often be the norm, especially among lower-ranked employees [49]. Thus, if one would assume that people’s tendency to act normatively in general is a correlate of 2D:4D (which is speculative, but possibly true), this could also explain the current pattern of findings.

### Limitations

A large number of 2D:4D studies have been published over the past years. How many (and which) of these studies can be replicated remains an open question [37,50,51]. Generally, though, there are some clear problems with 2D:4D research [51]: samples are typically small, effect sizes are typically modest, papers typically have only one study without a replication attempt, and analysis plans are typically not pre-registered, giving researchers much flexibility to stumble upon significant effects (even without malicious intent [52,53]). The present study suffers from all of these problems, and therefore, we cannot exclude the possibility that the present finding is a false positive. In particular, one limitation that needs to be highlighted is our sample size (N = 71), which is too small to attain stable parameter estimates in designs like ours [54,55]. Unfortunately, it was not possible to recruit more participants given the resources we had. While the findings of this study (p-values in particular) should thus be interpreted with caution, we included the data as supplementary information (S1 and S2 Files) and we hope that they will be re-used in the future (e.g., in pooled analyses).

Independently of the reproducibility issue mentioned in the previous paragraph, the quality of 2D:4D as a marker of prenatal testosterone has been subject to debate. For example, Berenbaum et al. [56] examined differences in 2D:4D between healthy men vs. men who are insensitive to the effects of testosterone (due to complete androgen insensitivity syndrome). In this study, the researchers noted large within-group variability and large between-group overlap. This pattern of results was in turn used to argue that 2D:4D is “not a good marker” (p. 5119) of individual differences in prenatal testosterone exposure. Based on a related line of reasoning, Apicella et al. [28] suggest that 2D:4D is “at the very best (…) an indirect and noisy measure” (p. 387). Yet, despite these conclusions, it may well be maintained that even though the association between 2D:4D and prenatal testosterone is too noisy to make classifications on the individual level, it is still the best available non-invasive, post-hoc marker of differences in prenatal testosterone exposure on the group level [57]. Since the present study examined people on the group level, we consider 2D:4D to be a satisfactory measure for the present purposes. It is important to note, though, that we do not wish to suggest that 2D:4D can be readily used in a test battery, e.g., for selecting employees.

A further limitation of this study is that we used a self-report instrument to measure people’s place in the organizational hierarchy. And, interestingly, 2D:4D has been reported to be associated with overconfidence in people’s own performance [58]. Although this association was not straightforward in previous research (its direction was context-dependent and it was found only among males) [58], it is possible that 2D:4D was related to people’s biased perception of their rank, rather than their actual rank. As we did not find evidence for such confounding in our study (2D:4D was not strongly related to place in the hierarchy; Table 1), we feel it is safe to assume that employees are capable of accurately estimating their place in the organizational hierarchy. Still, in future research, it would be advisable to use a more objective measure of rank.

A final limitation of this study is that we used self-report scales to measure voice [1]. While the scales were reliable, we cannot assume that all people self-reflect on their own voice behaviors in the same way, potentially challenging validity. In addition, as people may like to think of themselves as pro-active and honest people, it is possible that social desirability affected people’s responses. Speaking against the latter possibility, however, it should be noted that we did find a main effect of people’s place in the hierarchy. That is, high-rank (vs. low-rank) employees reported a stronger tendency to use voice. This finding is difficult to explain from a social-desirability perspective, as it seems unlikely that high-rank employees generally act in a more socially desirable way [59]. Nevertheless, it is important for future research to go beyond self-report measures of voice.

### Conclusion

The present study suggests that low-ranked employees who have been exposed to high concentrations of testosterone before birth, are less inclined to use voice behaviors within their organization. Perhaps, this is because prenatal exposure to testosterone causes people to strive to attain and maintain high social status later in life. In any case, by bridging management science and neuroendocrinology, this research suggests a new, biological way of thinking about people’s decisions to (not) use voice.

## Supporting information

S1 File Translated survey items. We used Dutch translations of existing questionnaires in this research. This MS Word document contains our translations.
(DOCX)

S2 File Data set. This MS Excel file contains the data we collected.
(XLSX)
