# Supplementary material for: Prenatal exposure to testosterone (2D:4D) and social hierarchy together predict voice behavior in bankers
Source: PLoS One. 2017 Jun 28;12(6):e0180008. doi: 10.1371/journal.pone.0180008 (PMC5489198; doi:10.1371/journal.pone.0180008)
Supplement: S1 File — We used Dutch translations of existing questionnaires in this research. This MS Word document contains our translations. (DOCX) [file pone.0180008.s001.docx]

**Supplementary document: Translated survey items**

In this research, we used Dutch translations of existing scales. This document contains these translations, along with the references to the original scales.

**Prohibitive voice**

**Translated from:** Liang J, Farh CI, Farh JL. Psychological antecedents of promotive and prohibitive voice: A two-wave examination. Academy of Management Journal. 2012;55: 71-92

within team

1. Ik spreek mijn collega’s aan op hun ongewenste gedrag dat de werkprestatie kan verminderen
2. Ik spreek problemen die mogelijk een nadeel voor het team kunnen veroorzaken uit, ondanks dat andere collega’s het niet met me eens zijn
3. Ik durf mijn mening uit te spreken over zaken die van invloed zijn op hoe efficiënt wij als team werken, zelfs als dit anderen zou beschamen
4. Ik wijs op problemen die ontstaan binnen het team, zelfs als dit mijn relatie met andere collega’s schaadt
5. Wanneer er binnen mijn team iets gebeurt dat nadelig zou kunnen zijn voor de organisatie, geef ik dit proactief aan bij mijn leidinggevende

within organization

1. Ik spreek collega’s in een ander organisatieonderdeel aan op hun ongewenste gedrag dat de werkprestatie kan verminderen
2. Ik spreek problemen die ik opmerk bij een ander organisatieonderdeel die mogelijk een nadeel voor de organisatie kunnen veroorzaken uit, ondanks dat collega’s het niet met me eens zijn
3. Ik durf mijn mening uit te spreken over zaken die van invloed zijn op hoe efficiënt er gewerkt wordt bij een ander organisatieonderdeel, zelfs als dit anderen zou beschamen
4. Ik wijs op problemen die ontstaan binnen de organisatie, zelfs als dit mijn relatie met andere collega’s schaadt
5. Wanneer ik opmerk dat er binnen een ander organisatieonderdeel iets gebeurt dat nadelig zou kunnen zijn voor de organisatie, geef ik dit proactief aan bij mijn leidinggevende of de leidinggevende van dat organisatieonderdeel

**BIS**

**Translated from:** Carver CS, White TL. Behavioral inhibition, behavioral activation, and affective responses to impending reward and punishment: The BIS/BAS scales. Journal of Personality and Social Psychology. 1994;67: 318-333.

1. Mijn collega’s zullen het mij niet kwalijk nemen als ik zorgen uitspreek over werkzaamheden, incidenten of gedrag van medewerkers dat nadelig is voor de organisatie.
2. Als ik zorgen uitspreek over werkzaamheden, incidenten of gedrag van medewerkers dat nadelig is voor de organisatie, zal hier iets mee gedaan worden.
3. Mijn leidinggevende zou het niet op prijs stellen als ik kritiek zou uiten over het functioneren van mijn team
4. Ik raak normaal gesproken behoorlijk gestrest als ik denk dat er iets vervelends te gebeuren staat
5. Ik maak me zorgen om fouten te maken
6. Kritiek of uitbranders raken mij behoorlijk
7. Ik voel me bezorgd of overstuur als ik denk of weet dat iemand boos op me is
8. Zelfs als me iets vervelends te wachten staat, voel ik me zelden angstig of nerveus
9. Ik voel me bezorgd als ik denk dat ik slecht heb gepresteerd
10. Vergeleken met mijn vrienden heb ik erg weinig angsten
11. Ik kan met anderen op het werk praten over wat ik echt belangrijk vind
12. Mijn taken op het werk stemmen overeen met wat ik echt wil doen
13. Ik voel niet echt een band met de andere mensen op mijn werk

**WRN-S**

**We used the original Dutch Scale from:** 26. Van den Broeck A, Vansteenkiste M, Witte H, Soenens B, Lens W. Capturing autonomy, competence, and relatedness at work: Construction and initial validation of the Work‐related Basic Need Satisfaction scale. Journal of Occupational and Organizational Psychology. 2010;83: 981-1002.

**Note:** We made some slight changes in wording because the original scale contained some words specific to Flemish-Dutch.

1. Ik voel me deel van een groep op mijn werk
2. Ik heb het gevoel dat ik ook de moeilijkste taken op mijn werk tot een goed einde kan brengen
3. Op mijn werk heb ik vaak het gevoel dat ik moet doen wat anderen mij bevelen
4. De mensen op mijn werk zijn echte vrienden
5. Ik heb de taken op mijn werk goed onder de knie
6. Ik voel me vrij mijn werk te doen zoals ik denk dat het goed is
7. Ik ga niet echt met de andere mensen op mijn werk om
8. Ik voel me bekwaam in mijn werk
9. Als ik mocht kiezen dan zou ik mijn werk anders aanpakken
10. Ik voel me vaak alleen als we onder collega’s zijn
11. Ik ben goed in mijn functie
12. Op mijn werk voel ik me gedwongen dingen te doen die ik niet wil doen
13. Ik heb het gevoel dat ik mezelf kan zijn op mijn werk
